# Mechanical Postconditioning Promotes Glucose Metabolism and AMPK Activity in Parallel with Improved Post-Ischemic Recovery in an Isolated Rat Heart Model of Donation after Circulatory Death

**DOI:** 10.3390/ijms21030964

**Published:** 2020-01-31

**Authors:** Maria Arnold, Natalia Méndez-Carmona, Patrik Gulac, Rahel K Wyss, Nina Rutishauser, Adrian Segiser, Thierry Carrel, Sarah Longnus

**Affiliations:** 1 Department of Cardiovascular Surgery, Inselspital, Bern University Hospital, 3010 Bern, Switzerland; 2 Department for BioMedical Research (DBMR), University of Bern, 3008 Bern, Switzerland; 3 Department of Pharmacology and Toxicology, Faculty of Pharmacy, Comenius University, 832 32 Bratislava 3, Slovakia

**Keywords:** cardiac ischemia reperfusion injury, donation after circulatory death, postconditioning, contractile function, glucose metabolism

## Abstract

Donation after circulatory death (DCD) could improve donor heart availability; however, warm ischemia-reperfusion injury raises concerns about graft quality. Mechanical postconditioning (MPC) may limit injury, but mechanisms remain incompletely characterized. Therefore, we investigated the roles of glucose metabolism and key signaling molecules in MPC using an isolated rat heart model of DCD. Hearts underwent 20 min perfusion, 30 min global ischemia, and 60 minu reperfusion with or without MPC (two cycles: 30 s reperfusion—30 s ischemia). Despite identical perfusion conditions, MPC either significantly decreased (low recovery = LoR; 32 ± 5%; *p* < 0.05), or increased (high recovery = HiR; 59 ± 7%; *p* < 0.05) the recovery of left ventricular work compared with no MPC (47 ± 9%). Glucose uptake and glycolysis were increased in HiR vs. LoR hearts (*p* < 0.05), but glucose oxidation was unchanged. Furthermore, in HiR vs. LoR hearts, phosphorylation of raptor, a downstream target of AMPK, increased (*p* < 0.05), cytochrome c release (*p* < 0.05) decreased, and TNFα content tended to decrease. Increased glucose uptake and glycolysis, lower mitochondrial damage, and a trend towards decreased pro-inflammatory cytokines occurred specifically in HiR vs. LoR MPC hearts, which may result from greater AMPK activation. Thus, we identify endogenous cellular mechanisms that occur specifically with cardioprotective MPC, which could be elicited in the development of effective reperfusion strategies for DCD cardiac grafts.

## 1. Introduction

For patients with end-stage heart failure, a heart transplantation is the gold-standard treatment [1]. Unfortunately, the supply of cardiac grafts is insufficient to cover the increasing demand [2]. Donation after circulatory death (DCD) could substantially increase donor heart availability; however, DCD hearts inevitably undergo warm, unprotected ischemia and reperfusion, which raise concerns about graft quality. Nonetheless, the feasibility of adult heart transplantation with DCD has been demonstrated in recent reports [3,4,5]. With DCD, increases in adult heart transplant activity of 15–33% have been observed in Australia and the UK since the implementation of DCD heart transplantation in their corresponding institutions [6,7]. As such, therapeutic strategies to optimize graft recovery following ischemia and reperfusion are now needed for the adoption of DCD heart transplantation in a safe and widespread manner. Because interventions prior to graft procurement are limited for ethical reasons in DCD, therapies, such as recruitment of endogenous protective mechanisms, at the time of reperfusion have the potential to provide considerable clinical impact by limiting graft ischemia-reperfusion injury.

Mechanical postconditioning (MPC; brief, intermittent periods of ischemia applied at the onset of reperfusion) can limit ischemia-reperfusion injury (IRI) by eliciting the heart’s endogenous protective mechanisms. Various reports have demonstrated the efficacy of MPC in limiting lethal reperfusion injury in experimental acute myocardial infarction [8,9,10]; however, clinical trials have been unable to demonstrate convincing protective effects [11]. On one hand, the lack of effective clinical cardioprotection may result from technical aspects, such as inability to implement conditioning in a timely manner, as well as patient co-morbidities and co-medications that interfere with the effective recruitment of conditioning mechanisms [11]. In DCD, reperfusion is a clinically scheduled, timely intervention and co-morbidities and co-medications are limited through donor selection. Therefore, approaches such as MPC may provide more consistent protection in this context. On the other hand, the cardioprotective efficacy of MPC varies according to the experimental model and specific conditions (i.e., energy substrate availability [12], age [13], in vivo vs. ex vivo [14], Langendorff vs. working mode [15], ischemic temperature [15] or postconditioning algorithm [16]). This variability highlights our incomplete understanding of the precise mechanisms responsible for the cardioprotective effects of MPC. As such, its value may lie in its use as an experimental tool for the identification of endogenous cardiac mechanisms as targets amenable to pharmacological intervention, rather than as a potential therapeutic approach.

MPC has been reported to provide cardioprotection through several mechanisms. The reperfusion injury salvage kinase (RISK) pathway, comprising the phosphoinositide 3-kinase (PI3K)/Akt and extracellular signal-regulated kinase (Erk) 1/2 pro-survival kinase cascades, as well as the survivor activating factor enhancement (SAFE) pathway may attenuate reperfusion-induced cell death via the preservation of mitochondrial integrity [17,18]. 5′AMP-activated protein kinase (AMPK) has also been proposed to contribute to some MPC-induced cardioprotective effects; however, it has not been clearly designated as a mediator of MPC, as its activity has not been evaluated [19]. MPC may also provide cardioprotection via the reduction in pro-inflammatory responses by reducing accumulation of neutrophils in the myocardial infarction zone [20], and/or decreasing plasma tumor necrosis factor alpha (TNFα) levels [21]. Interestingly, AMPK also protects against TNFα-induced cardiac cell death [22] and reduces macrophage inflammation [23]; however, its role in anti-inflammatory effects induced by MPC-induced cardioprotection has not been investigated.

Despite the fact that energy metabolism plays a fundamental role in IRI and may be affected by the signaling pathways stimulated by MPC, few investigators have addressed the role of energy metabolism in MPC-induced cardioprotection. Reperfusion of an ischemic area typically results in the rapid recovery of fatty acid oxidation, which inhibits glucose use, particularly its oxidation [24]. Importantly, in DCD, as well as in other clinical situations of warm ischemia-reperfusion, the heart is exposed to high levels of free fatty acids prior to warm ischemia [25], which can be detrimental for post-ischemic function [26]. Shifting energy substrate metabolism away from fatty acid oxidation and towards glucose use during early reperfusion has been proposed to limit IRI and improve subsequent contractile recovery [27]. Interestingly, activation of Akt and AMPK, both implicated in MPC-induced cardioprotection, would be expected to promote glucose metabolism [28,29]; however, these mechanisms have not yet been investigated.

We therefore aimed to investigate the effects of MPC on glucose metabolism and its key regulatory pathways during early reperfusion, in order to better characterize endogenous mechanisms of cardioprotection in an isolated, working rat heart model of DCD that includes clinically relevant circulating levels of free fatty acids prior to warm ischemia.

## 2. Results

### 2.1. Baseline Characteristics

Baseline characteristics and sample sizes for experimental Series A and B are shown in Table 1. Within each series, no difference among the experimental groups was observed. 

### 2.2. Post-Ischemic Cardiac Functional Recovery

**Figure 1:**
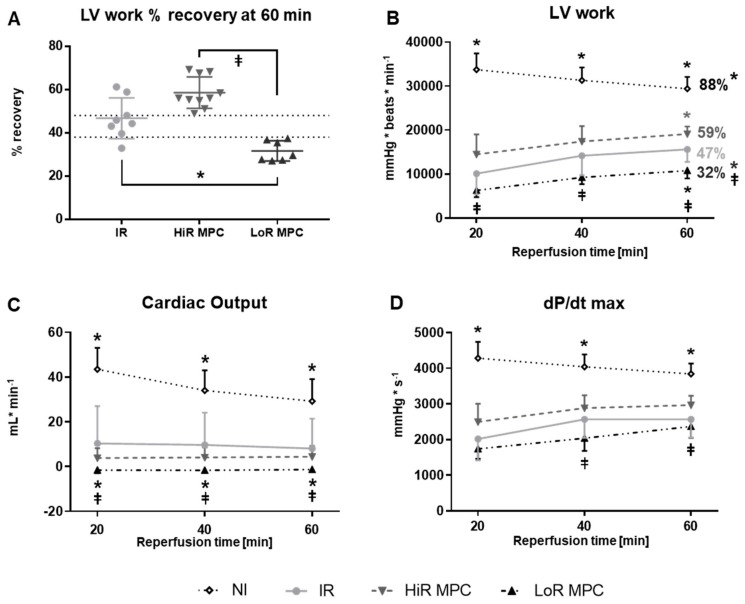
Post-ischemic contractile recovery. (**A**) Distribution of left-ventricular work (LV work = developed pressure × heart rate [mmHg × beats × min^−1^]) recovery (LV work value at 60 min reperfusion expressed as a percentage of mean baseline value). Two MPC subgroups were observed: high (HiR) and low (LoR) recovery; (**B**) LV work (percentages: LV work values at 60 min reperfusion expressed as a percentage of mean baseline value); (**C**) Cardiac output; (**D**) dP/dt max (maximum first derivative of LV pressure). IR, ischemia reperfusion; MPC, mechanical postconditioning; NI, no ischemia. Data are expressed as mean ± standard deviation. * *p* < 0.05 vs. IR, ⱡ *p* < 0.05 LoR MPC vs. HiR MPC (*n* = 7–11/group).

Among hearts that underwent ischemia, those that were treated with MPC demonstrated substantially more variability during recovery than those ischemic hearts without treatment (IR). More specifically, MPC hearts, despite the same protocol, tended to recover relatively well (>48% left ventricular (LV) work recovery) or rather poorly (<38%; Figure 1A). The clear separation in recovery observed in hearts subjected to MPC allowed us to designate two recovery subgroups: MPC high-recovery (HiR) and low-recovery (LoR) subgroups, based on a cut-off value of 45% LV work recovery at 60 min reperfusion (or 25% LV work recovery at 15 min reperfusion).

Absolute values of cardiac functional parameters during reperfusion are represented in Figure 1B–D. As expected, post-ischemic cardiac function was significantly decreased in IR hearts compared to NI hearts in terms of LV work, cardiac output, dP/dt max (*p* < 0.05; Figure 1B–D) as well as heart rate, developed pressure and dP/dt min (*p* < 0.05; data not shown), but not coronary flow (data not shown). MPC significantly increased (HiR) or decreased (LoR) left ventricular work at 60 min reperfusion compared to IR hearts (*p* < 0.05 for both; Figure 1B). Significant differences between HiR and LoR MPC hearts were observed for LV work and cardiac output at all time points, and for dP/dt max at 40 and 60 min reperfusion (all *p* < 0.05; Figure 1B–D).

### 2.3. Markers of Cell Damage

**Figure 2:**
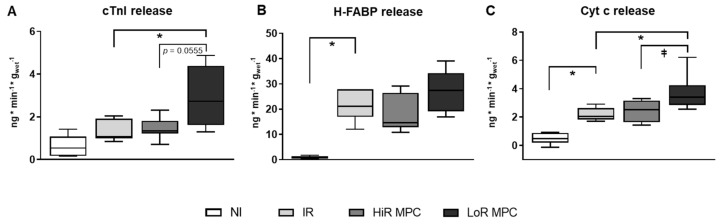
Release of circulating markers of cell death and mitochondrial damage at 10 min reperfusion. (**A**) cardiac troponin I (cTnI); (**B**) heart-type fatty acid binding protein (H-FABP); (**C**) cytochrome c (Cyt c). HiR, high recovery; IR, ischemia reperfusion; LoR, low recovery; MPC, mechanical postconditioning; NI, no ischemia. Data are expressed as median, 25–75 percentiles and range. * *p* < 0.05 vs. IR, ⱡ *p* < 0.05 LoR MPC vs. HiR MPC (n = 6–10/group).

Markers of cellular (cardiac troponin I (cTnI) and heart-type fatty acid binding protein (H-FABP)) and mitochondrial (cytochrome c (cyt c)) damage were measured at 10 min reperfusion. Release of cTnI, H-FABP and cyt c appeared greater in IR vs. NI hearts, but reached statistical significance only for H-FABP and cyt c (*p* < 0.05 for both, *p* = 0.1104 for cTnI; Figure 2). LoR MPC hearts, but not HiR MPC hearts, released more cyt c and cTnI compared to IR (*p* < 0.05 for both). Furthermore, a significantly greater cyt c release (*p* < 0.05) as well as a tendency for a greater cTnI and H-FABP release (*p* = 0.0555 and *p* = 0.1293 respectively) were observed in LoR vs. HiR MPC hearts.

### 2.4. Post-Ischemic Metabolic Recovery

**Figure 3:**
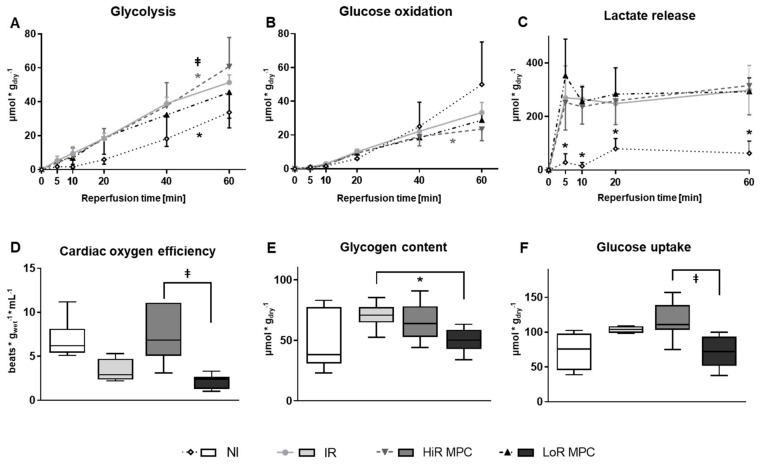
Post-ischemic metabolic recovery. (**A**) Rates of glycolysis; (**B**) Rates of glucose oxidation; (**C**) Lactate accumulation (net change) in recirculating perfusate; (**D**) Oxygen efficiency [LV work/oxygen consumption] at 15 min reperfusion; (**E**) Glycogen content at 60 min reperfusion; (**F**) Glucose uptake (calculated) at 60 min reperfusion. HiR, high recovery; IR, ischemia reperfusion; LoR, low recovery; MPC, mechanical postconditioning; NI, no ischemia. Data are expressed as mean ± standard deviation (**A**–**C**) or as median, 25–75 percentiles and range (**D**–**F**). * *p* < 0.05 vs. IR, ⱡ *p* < 0.05 LoR MPC vs. HiR MPC (*n* = 4–11/group).

Higher rates of glycolysis during the 60 min reperfusion period were observed in IR compared to NI hearts (*p* < 0.05). Among hearts subjected to ischemia, glycolysis rates were highest in HiR hearts (*p* < 0.05 vs. IR and vs. LoR; Figure 3A). Glucose oxidation rates during reperfusion on the other hand, were significantly decreased in HiR MPC vs. IR hearts (*p* < 0.05; Figure 3B), but not different in LoR MPC vs. IR hearts.

As expected, less lactate was released in NI vs. ischemic hearts (*p* < 0.05 vs. IR) at all reperfusion time points (Figure 3C). No significant differences were observed among hearts subjected to ischemia.

Oxygen efficiency, the ratio of LV work to oxygen consumption, tended to be lower in IR vs. NI hearts (*p* = 0.0570), and was significantly lower in LoR vs. HiR MPC hearts (*p* < 0.05; Figure 3D).

Glycogen content at the end of reperfusion was lower in LoR MPC hearts compared to IR (*p* < 0.05; Figure 3E) and glucose uptake was decreased in LoR vs. HiR MPC hearts (*p* < 0.05; Figure 3F).

### 2.5. Intracellular Signaling Pathways

**Figure 4:**
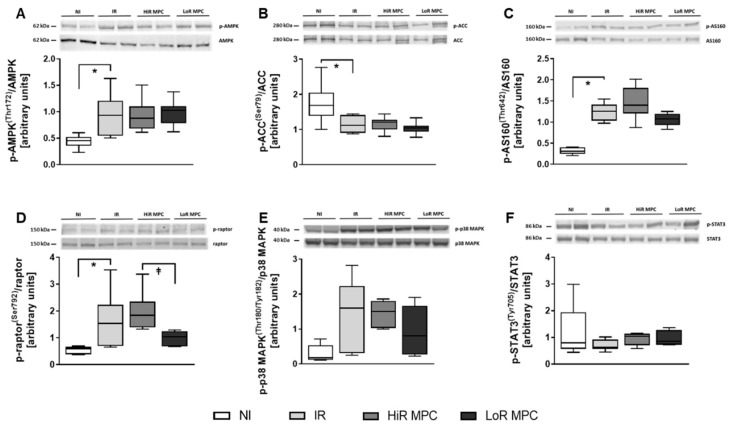
Activation of key signaling molecules—AMPK and Survivor Activating Factor Enhancement (SAFE) pathway. Representative Western blots for phosphorylated and total proteins in top panels and ratios of phosphorylated to total proteins for: (**A**) AMP-activated protein kinase (AMPK) [Thr172]; (**B**) acetyl-CoA carboxylase (ACC) [Ser79]; (**C**) Akt substrate of 160 kDa (AS160) [Thr642]; (**D**) regulatory associated protein of mTOR (raptor) [Ser792]; (**E**) p38 mitogen-activated protein kinase (p38 MAPK) [Thr180/Tyr182]; (**F**) signal transducer and activator of transcription 3 (STAT3) [Tyr705]. HiR, high recovery; IR, ischemia reperfusion; LoR, low recovery; MPC, mechanical postconditioning; NI, no ischemia. Data are expressed as median, 25–75 percentiles and range. * *p* < 0.05 vs. IR, ⱡ *p* < 0.05 LoR MPC vs. HiR MPC (*n* = 5–10/group).

**Figure 5:**
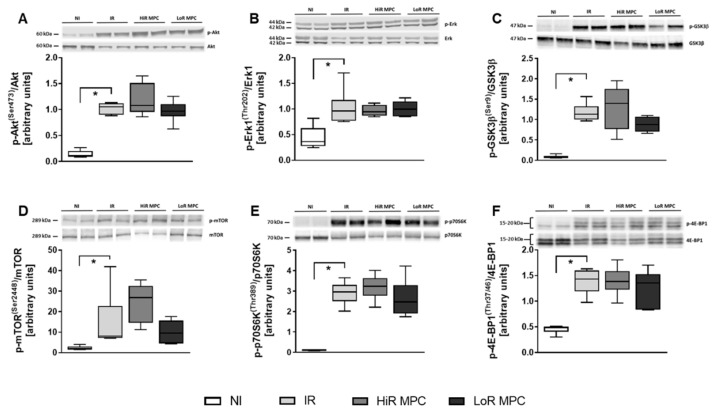
Activation of key signaling molecules—Reperfusion Injury Salvage Kinase (RISK) and mTOR pathway. Representative Western blots for phosphorylated and total proteins in top panels and ratios of phosphorylated to total proteins for: (**A**) Akt [Ser473]; (**B**) extracellular signal-regulated kinase 1 (Erk1) [Thr202]; (**C**) glycogen synthase kinase 3 beta (GSK3β) [Ser9]; (**D**) mammalian target of rapamycin (mTOR) [Ser2448]; (**E**) ribosomal protein S6 kinase (p70S6K) [Thr389]; (**F**) eukaryotic translation initiation factor 4E-binding protein 1 (4E-BP1) [Thr37/46]. HiR, high recovery; IR, ischemia reperfusion; LoR, low recovery; MPC, mechanical postconditioning; NI, no ischemia. Data are expressed as median, 25–75 percentiles and range. * *p* < 0.05 vs. IR (*n* = 4–7/group).

Western blots were performed at 15 min reperfusion to investigate the activation of key signaling pathways during early reperfusion. AMPK phosphorylation was increased in ischemic hearts (*p* < 0.05 IR vs. NI), but was not different between IR, HiR and LoR MPC (Figure 4A). Phosphorylation of various downstream targets of AMPK are presented in Figure 4B-E (ACC, AS160, raptor, p38 MAPK). Raptor phosphorylation was significantly higher in HiR vs. LoR MPC hearts (*p* < 0.05), and although not statistically significant, AS160 demonstrated a tendency for greater phosphorylation in HiR vs. LoR MPC hearts (*p* = 0.077). As a representative of the SAFE pathway, STAT3 phosphorylation was measured. No differences were observed between experimental groups (Figure 4F). Upon reperfusion following ischemia, molecules of the RISK (Akt, Erk1, GSK3β) and mTOR pathways (mTOR, p70S6K, 4E-BP1) demonstrated significantly greater phosphorylation than those without ischemia (*p* < 0.05 IR vs. NI for all; Figure 5). No significant differences in the phosphorylation pattern of these molecules could be observed between IR, HiR MPC and LoR MPC. However, a tendency for greater mTOR phosphorylation could be observed in HiR vs. LoR MPC hearts (*p* = 0.078).

### 2.6. Cytokine Content

**Figure 6:**
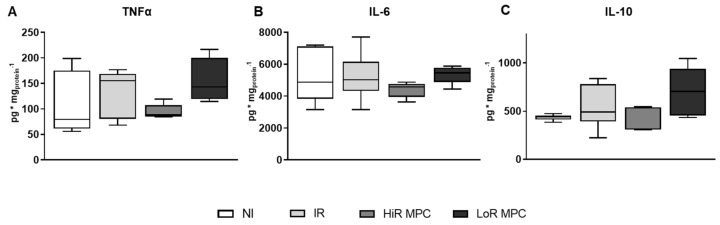
Cardiac cytokine content at 15 min reperfusion. (**A**) Tumor necrosis factor alpha (TNFα); (**B**) interleukin 6 (IL-6); (**C**) interleukin 10 (IL-10). HiR, high recovery; IR, ischemia reperfusion; LoR, low recovery; MPC, mechanical postconditioning; NI, no ischemia. Data are expressed as median, 25–75 percentiles and range. (*n* = 5–8/group).

The cardiac content of pro- and anti-inflammatory cytokines was determined at 15 min reperfusion (Figure 6). TNFα and interleukin-6 (IL-6) levels tended to be decreased in HiR MPC hearts compared to LoR MPC hearts.

## 3. Discussion

Using an isolated rat heart model of DCD, we demonstrate that MPC can have deleterious—as well as protective—effects on post-ischemic cardiac recovery, and is therefore not a safe strategy to use with DCD cardiac grafts. Importantly, the emergence of HiR and LoR MPC subgroups, with significantly different contractile recoveries despite identical treatment, could help to identify endogenous, protective mechanisms that are specifically associated with cardioprotection. Indeed, MPC reduced functional recovery, induced greater mitochondrial damage, and increased cell death in LoR hearts. However, MPC improved functional recovery while increasing rates of glycolysis and reducing glucose oxidation in HiR hearts. In parallel with improved contractile function in HiR vs. LoR MPC hearts, glucose uptake, glycolysis, mitochondrial preservation, and oxygen efficiency were increased. Our findings support a role for AMPK activation in MPC-induced protective effects, likely via the phosphorylation of AS160, in increasing glucose uptake and glycolysis. Furthermore, reduced levels of circulating cyt c and tendencies for lower cardiac TNFα in HiR vs. LoR MPC hearts are consistent with a mechanism involving AMPK activation. Taken together, we identify mechanisms that occur specifically with MPC only when it provides cardioprotection, which could be used towards the development of pharmacologic targets for safe, effective, and reliable reperfusion strategies to help ensure optimal function of DCD cardiac grafts.

Conditioning of the heart after the onset of ischemia, for example: MPC or remote conditioning, is particularly attractive in settings when ischemia cannot be anticipated, and has previously been investigated in the context of myocardial infarction. Many pre-clinical studies to ‘condition’ the heart have demonstrated successful protection against ischemia-reperfusion injury. However, the cardioprotective efficacy of MPC varies according to the experimental model and specific conditions [12,13,14,15,16] and translation into clinics has not yet been successful [11,30]. Just recently, an international randomized controlled trial on the effect of remote ischemic conditioning (CONDI-2/ERIC-PPCI trial) in patients with acute myocardial infarction reported no improvement in clinical outcome [31]. These recent findings further stress our incomplete understanding of cardiac conditioning mechanisms. Problematic issues mentioned for the translatability of pre-clinical studies, as timely intervention or co-medications [11], will be less of a problem in a DCD setting compared to myocardial infarction. In our study, with the emergence of high and low subgroups with MPC, we can identify MPC-induced effects that are specifically associated with improved cardiac recovery, thereby providing new mechanistic information. We reveal that AMPK activation (indicated by greater raptor phosphorylation) was prolonged and glucose metabolism (especially glucose uptake and glycolysis) was stimulated by MPC only when functional recovery increased, and not when functional recovery decreased. As such, the pharmacologic stimulation of AMPK and glucose metabolism during DCD heart procurement might be a promising approach for limiting IRI. In human DCD heart transplantation, pharmacologic cardioprotection is already used—erythropoietin and the nitric oxide donor glyceryl trinitrate are provided in the cardioplegia [3]. Therefore, the pharmacologic stimulation of AMPK and glucose metabolism (for example, with metformin [32]) could be complementary and would be interesting to investigate. Importantly, in clinical DCD protocols, the heart will be reperfused (isolated) on an *ex-situ* perfusion machine [3] and thus, the potential detrimental impact of drugs or drug doses on other organ systems will be limited.

MPC reduced recovery in some hearts (LoR MPC) compared with hearts subjected to ischemia and reperfusion without MPC. In these hearts, functional recovery decreased, while indicators of mitochondrial damage and cell death increased, consistent with reperfusion-injury-induced mitochondrial damage and subsequent cell death [33,34]. Interestingly, activation of the RISK pathway (Akt and Erk) was increased during early reperfusion in all hearts exposed to ischemia (compared to non-ischemic controls), but was not further elevated by the application of MPC, suggesting that conditioning mechanisms that are dependent on the RISK pathway are already maximally elicited under these conditions, and cannot be further stimulated by MPC. Thus, we cannot attribute the lack of cardioprotection in LoR MPC hearts to ineffective RISK pathway activation. In our study, hearts underwent 30 min normothermic, global ischemia, which is severe, but corresponds with the maximal warm ischemic time permitted in clinical DCD heart transplantation protocols [35]. Under these conditions, it would be reasonable to speculate that, for some hearts, we have exceeded the ischemic time window for which MPC is able to provide effective cardioprotection, and that the additional short intervals of ischemia during MPC exacerbate IRI.

However, in other hearts, MPC improved recovery after ischemia compared with non-MPC-treated hearts. Contractile recovery increased in HiR MPC vs. IR hearts in parallel with changes in glucose metabolism. Our findings of greater glucose uptake and glycolysis are consistent with those of Correa et al., who reported increases in glycolysis and GLUT4 translocation during early reperfusion in association with MPC-induced cardioprotective effects in isolated rat hearts [36]. Furthermore, we observed a decrease in glucose oxidation in HiR MPC vs. IR hearts during reperfusion. To our knowledge, effects of MPC have not previously been reported on glucose oxidation. The increased glycolysis but lower glucose oxidation rates in hearts with higher recovery suggests greater uncoupling between glycolysis and glucose oxidation in HiR MPC hearts, which is rather unexpected, as this results in the accumulation of intracellular protons [37]. This accumulation could lead to a decreased cardiac function, as more ATP would be needed for normalization of intracellular pH and therefore less ATP will be available for myocardial contraction [37]. On the other hand, increased glycolysis may be beneficial as glycolytically-derived ATP plays a critical role in maintaining calcium homeostasis during reperfusion [38], and thereby helps to prevent intracellular calcium overload, which could induce cardiomyocyte death by hypercontracture or mitochondrial damage [34]. Taken together, it appears that increased glucose uptake and glycolysis occur in parallel with MPC-induced improvements in contractile recovery during early reperfusion; however, precisely how these alterations influence cardiac recovery remains to be determined.

In our study, MPC did not consistently provide cardioprotection, but resulted in the emergence of recovery subgroups. The precise reason underlying differing responses to MPC, despite identical treatment, remains unclear. Since no subgroups were observed in NI or IR groups, we can exclude artefacts arising from the isolated heart preparations or perfusion technique. In a similar model, it has been reported that subgroups can occur during reperfusion and that these subgroups depend on energy substrate availability [12]. A role for energy metabolism in the development of subgroups is consistent with our findings of differing glucose metabolism in HiR vs. LoR MPC hearts. Notably, the existence of two subgroups in our study provides the unique opportunity to directly compare high- vs. low-recovery hearts that were all subjected to MPC, and may therefore aid in pinpointing critical MPC-induced protective mechanisms, while eliminating MPC-associated changes that do not contribute to cardioprotection.

**Figure 7:**
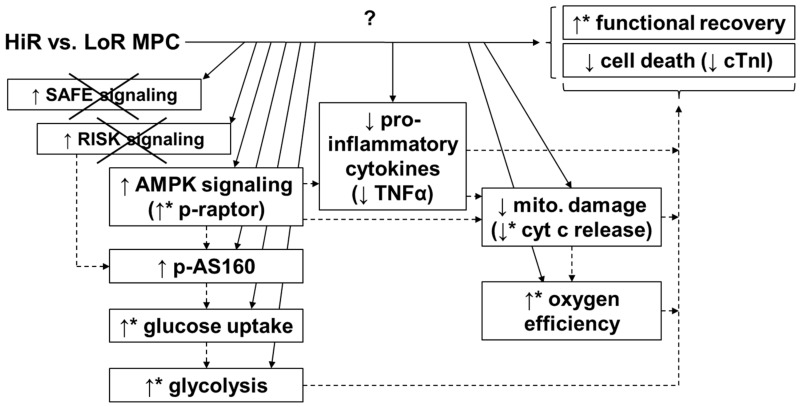
Potential mechanism for MPC-induced cardioprotection. HiR MPC hearts presented better functional post-ischemic recovery, as well as reduced release of cell death markers and lower tissue levels of pro-inflammatory cytokines, compared with LoR MPC hearts. Increased AMPK activation (raptor and AS160), greater glucose uptake and glycolysis, and less mitochondrial damage may all contribute to the cardioprotective effects of MPC. No evidence for the involvement of the SAFE or RISK pathway was observed (as indicated by the “x” in the figure) under these conditions. ↑: higher in HiR vs. LoR MPC hearts. ↓: lower in HiR vs. LoR MPC hearts. ↑ or ↓with *: *p* < 0.05 between HiR and LoR MPC hearts, ↑ or ↓ without * indicate non-significant tendency between HiR and LoR MPC hearts. Arrows (continuous lines) connecting boxes: demonstrated relationship. Arrows (dashed lines) connecting boxes: expected/proposed relationship. AMPK, AMP-activated protein kinase; AS160, Akt substrate of 160 kDa; cTnI, cardiac troponin I; cyt c, cytochrome c; HiR, high recovery; LoR, low recovery; MPC, mechanical postconditioning; raptor; regulatory associated protein of mTOR; RISK, reperfusion injury salvage kinase; SAFE, survivor activating factor enhancement; TNFα, tumor necrosis factor alpha.

Differences between high and low recovery subgroups may be particularly useful for improving our understanding of cardioprotective mechanisms (Figure 7). In our study, glucose uptake and glycolysis were significantly higher in HiR vs. LoR MPC hearts. One mechanism for increased glucose uptake and glycolysis is through activation of AMPK [39], which has been implicated in MPC, but not clearly designated as a mediator, as its activity was not evaluated [19]. In our study, AMPK phosphorylation levels between HiR and LoR MPC groups were similar at 15 min reperfusion, but it is important to note that AMPK phosphorylation decreases rapidly in this model upon reperfusion (unpublished data). As such, and given the possibility of allosteric activation of AMPK [40], downstream targets of AMPK were also evaluated. Greater phosphorylation of raptor, a direct substrate of AMPK [41], in HiR vs. LoR MPC hearts supports the concept that AMPK activation is prolonged after ischemia in HiR MPC, but not LoR MPC hearts. AMPK can phosphorylate AS160 [29], promoting the translocation of GLUT4 to the plasma membrane and stimulating glucose uptake [42]. Phosphorylation of the Rab GTPase-activating protein AS160 (also known as TBC1D4) also tended to increase in HiR vs. LoR MPC hearts. To our knowledge, MPC-induced activation of AS160 has not been reported. Akt can also phosphorylate AS160 [29], however, we did not observe any difference in Akt phosphorylation or its downstream targets between HiR and LoR MPC groups. It is thus tempting to speculate that the increased AS160 phosphorylation in HiR vs. LoR MPC hearts underlies increases in glucose uptake and glycolysis downstream of AMPK in our study.

Mitochondria were better preserved in HiR vs. LoR MPC hearts, as indicated by lower cyt c release. Consistent with these findings, HiR MPC hearts were more oxygen-efficient (greater LV work performed per oxygen consumed) than LoR MPC hearts during early reperfusion. Importantly, MPC can inhibit irreversible opening of the mitochondrial permeability transition pore (mPTP), which is associated with the release of cytochrome c and the collapse of the membrane potential and leads to cell death [43,44]. We have previously reported that MPC-induced cardioprotection is associated with improved mitochondrial function (greater complex I activity and ATP content and less cyt c release) in a similar model, therefore supporting the concept of MPC-induced mitochondrial preservation [45]. Interestingly, evidence exists to support a role for AMPK in the inhibition of mPTP opening [32], which is consistent with a role for AMPK in the cardioprotection observed in HiR MPC hearts. When phosphorylated, GSK3β, a downstream target of Akt and Erk, inhibits the opening of the mPTP [46]. Even though mitochondrial damage was reduced in HiR vs. LoR MPC hearts, as indicated by lower cyt c release, this was not reflected by GSK3β phosphorylation, suggesting that mitochondrial preservation in HiR MPC hearts was not mediated by the RISK pathway. Additionally, we measured STAT3 phosphorylation as a representative of the SAFE pathway. Phosphorylation was not different among experimental groups, indicating that SAFE pathway activation is not involved in the improved recovery observed in HiR vs. LoR MPC hearts.

As the perfusate levels of pro- and anti-inflammatory cytokines are expected to be low to undetectable in our experimental setup [47], we have determined their cardiac content. TNFα tissue content tended to be reduced in HiR compared to LoR MPC hearts. TNFα is part of the innate immune system and also initiates cardioprotective signaling pathways. Briefly, low TNFα doses limit IRI, whereas higher TNFα doses increase infarct size when given before ischemia [48]. Interestingly, AMPK also plays an acute anti-inflammatory role [23]. Reduced TNFα content in HiR vs. LoR MPC hearts further supports greater AMPK activation in HiR MPC hearts. Finally, the induction of mPTP opening and release of cytochrome c by TNFα has been reported previously [49]. This is consistent with our findings of a lower cytochrome c release and lower TNFα content at early reperfusion in HiR vs. LoR MPC hearts. Moreover, TNFα can initiate the SAFE pathway [50]; however, differing TNFα in HiR and LoR MPC hearts was not reflected in STAT3 phosphorylation levels.

This study does possess some limitations. Our experimental (*ex-situ*) model and the specific conditions were chosen in order to limit variability and strictly control the energy substrate availability and duration and temperature of ischemia to simulate DCD conditions; however, it does not incorporate the physiologic changes in the body following withdrawal of life sustaining therapy (typical DCD heart donor), such as the catecholamine storm or pulmonary vasoconstriction leading to a pressure-volume overload in the right heart [51,52]. Furthermore, although we provide new information about potential mechanisms of MPC-mediated cardioprotection, additional studies are required to fully characterize the precise molecular causes of MPC-induced effects. For example, specific AMPK inhibition at reperfusion onset used in parallel with MPC or studies in hypoxia/reoxygenation cell culture models could help to elucidate the cardioprotective effects of MPC. Pressure-volume loop analysis was not used in this study and could add further information about heart function.

## 4. Materials and Methods

### 4.1. Ethics Statement

All experimental procedures were carried out in compliance with the European Convention for Animal Care and approved by the Swiss animal welfare authorities and the Ethics Committee for Animal Experimentation, Berne, Switzerland (Veterinärdienst des Kantons Bern; BE59/14; approval: 18.08.2014). Surgery was performed under general anesthesia, and all efforts were made to minimize animal suffering.

### 4.2. Isolated Heart Perfusions

**Figure 8:**
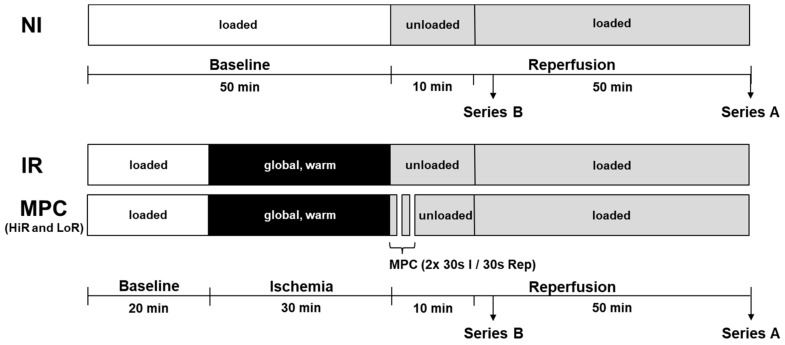
Experimental protocol. Hearts underwent 20 min of baseline, working-mode (loaded) perfusion, followed by either 0 or 30 min of warm, global ischemia, and reperfusion (10 min unloaded followed by working-mode (loaded) perfusion). In the treatment group, mechanical postconditioning (MPC) was performed immediately following ischemia (two cycles of 30 s reperfusion/30 s ischemia), which led to the emergence of two subgroups, referred to as HiR and LoR MPC. In Perfusion Series A, hearts were reperfused for a total of 60 min for the measurement of contractile and metabolic recovery (glycolysis, glucose oxidation, lactate, glycogen, glucose uptake). In Perfusion Series B, hearts were treated identically, but were stopped after 15 min reperfusion for the analysis of key intracellular signaling pathways. Circulating markers of mitochondrial and cellular damage, as well as oxygen efficiency were also measured in this series of hearts. HiR, high recovery; IR, ischemia reperfusion; LoR, low recovery; NI, no ischemia.

An isolated heart model of DCD heart transplantation was used, as previously reported [26,45,47,53,54], with male Wistar rats (Janvier Labs, Le Genest-Saint-Isle, France) of 10 to 11 weeks of age to ensure mature cardiac metabolism [55] and to represent young, adult human DCD donors. Rats were housed under standard conditions at a controlled room temperature with a 12-h light–dark cycle and access to water and food *ad libitum*. After random assignment to experimental groups (in alternating fashion), the rats were anaesthetized intraperitoneally using 100 mg/kg ketamine (Narketan^®^, Vetoquinol AG, Bern, Switzerland) and 10 mg/kg xylazine (Xylapan^®^, Vetoquinol AG, Bern, Switzerland). As soon as the pedal reflex had disappeared, the hearts were excised and cannulated on the perfusion system. Series A: Hearts first underwent 20 min of baseline working mode perfusion with modified KHB buffer (containing 118 mM NaCl, 4.7 mM KCl, 1.2 mM KH_2_PO_4_, 1.25 mM CaCl_2_·2H_2_O, 1.2 mM MgSO_4_·7H_2_O, 25 mM NaHCO_3_ and 11 mM glucose, supplemented with 1.2 mM palmitate/3% BSA and 0.5 mM lactate; gassed with 95% O_2_- 5% CO_2_). Thereafter, hearts were subjected to 30 min global, no-flow, normothermic (37 °C) ischemia. The first ten minutes of reperfusion following ischemia were performed in Langendorff/unloaded mode; subsequently the hearts were switched to working mode for the following 50 min reperfusion (reperfusion buffer: modified KHB buffer without addition of palmitate/BSA and lactate; Figure 8). Following ischemia, hearts were either subjected to mechanical postconditioning (MPC; two cycles of 30 s reperfusion/30 s ischemia), or directly reperfused (ischemia reperfusion, IR). In addition, a non-ischemic control group (no ischemia, NI) was generated by prolonging the aerobic, baseline perfusion conditions for the equivalent time of ischemia, followed by reperfusion as described above. Series B: Hearts were perfused using the three protocols described above, but stopped after a total of 15 min reperfusion for molecular analyses. For glycogen content measurements, an additional group of hearts was stopped directly after ischemia.

Buffer samples from preload and coronary effluent were regularly taken throughout the perfusion protocol and stored at −20 °C until analyzed.

As soon as the perfusion protocol was finished, perfusate lines to the heart were clamped and the heart was immediately frozen with Wollenberger clamps cooled in liquid nitrogen. The heart was then put into liquid nitrogen and stored at −80 °C until needed.

### 4.3. Functional Data Collection

To obtain data for cardiac function, a micro-tip pressure catheter (Millar, Houston, TX, USA) placed in the left ventricle was used to continuously measure heart rate (HR), peak systolic pressure, developed pressure (DP), minimum and maximum first derivatives of left ventricular pressure (dP/dt min and dP/dt max). Left ventricular work (LV work) was calculated as previously described [45,47,53,54]: DP × HR.

Flowmeters (Transonic Systems Inc., Ithaca, NY, USA) were used to measure perfusate flows in preload and afterload lines, to determine coronary flow and cardiac output.

All data were recorded using a PowerLab data acquisition system (ADInstruments, Spechbach, Germany).

### 4.4. Rates of Glycolysis and Glucose Oxidation

Radiolabeled glucose ([5-^3^H]glucose and [U-^14^C ]glucose; PerkinElmer, Waltham, MA, USA) was added into the reperfusion buffer in an air-tight perfusion system with a hyamine hydroxide CO_2_ trap. Buffer samples were taken at various reperfusion time points and stored at −20°C until analyzed. Protocols for the measurement of glycolysis and glucose oxidation are described elsewhere [26,56]. Wet/dry weight ratio was used to normalize values.

### 4.5. Cytochrome c, Cardiac Troponin I and Heart-Type Fatty Acid Binding Protein

10 min reperfusion buffer samples from Series B were used for ELISA measurements of cyt c (R&D Systems, Minneapolis, MN, USA), cTnI (Life Diagnostics, West Chester, PA, USA) and H-FABP (Life Diagnostics, West Chester, PA, USA). For all of these measurements, cardiac release was calculated as:

(1) $$\frac{C\left(t\right)\times V_{t}}{T\times HW}$$

C = concentration in recirculating buffer, HW = heart weight, t = time point of interest, V = buffer volume, T = perfusion duration

### 4.6. Lactate

Buffer samples from 0, 5, 10, 20, and 60 min reperfusion from Series A were measured with a lactate assay kit (Sigma-Aldrich, St. Louis, MO, USA). Lactate accumulation was calculated as:

(2) $$\frac{C\left(t\right)\times V_{t}-C\left(t_{0}\right)\times V_{0}}{HW}$$

C = concentration in recirculating buffer, HW = heart weight, t = time point of interest, V = buffer volume, Wet/dry weight ratio was used to normalize.

### 4.7. Cardiac Oxygen Consumption and Oxygen Efficiency

Oxygen consumption was determined using the Cobas b 123 blood-gas analyzer (Roche, Basel, Switzerland). Cardiac oxygen consumption was calculated as:

(3) $$\frac{\left(C_{CE}\left(t\right)-C_{PL}\left(t\right)\right)\times CF\left(t\right)}{HW}$$

Cardiac oxygen efficiency was calculated as:

(4) $$\frac{LV\;work\;\left(t\right)}{O_{2}C\;\left(t\right)}$$

C = concentration, CE = coronary effluent, CF = coronary flow, HW = heart weight, LV work = left ventricular work, O_2_C = oxygen consumption, PL = preload, t = time point of interest

### 4.8. Glycogen Content

Myocardial glycogen tissue content was determined using a spectrophotometric glucose assay kit (Sigma-Aldrich, St. Louis, MO, USA) as previously described [26,57]. Wet/dry weight ratio was used to normalize.

### 4.9. Glucose Uptake

Glucose uptake over the 60 min reperfusion period was calculated at as the sum of glucose that passed through glycolysis and glucose that was incorporated into glycogen (glycogen (60 min) − glycogen (mean end-ischemia)).

### 4.10. Phosphorylation of Key Signaling Molecules

In order to measure the phosphorylation status of key signaling molecules, Western blots were performed according to the protocol described earlier [47]. The following antibodies were obtained from Santa Cruz Biotechnology (Dallas, TX, USA): anti-GSK3β and anti-IRβ, others from Cell Signaling Technology (Danvers, MA, USA): anti-AMPKα, anti-phospho-(Thr172)-AMPKα, anti-ACC, anti-phospho-(Ser79)-ACC, anti-Akt, anti-phospho-(Ser473)-Akt, anti-AS160, anti-phospho-(Thr642)-AS160, anti-phospho-(Ser9)-GSK3β, anti-ERK1/2, anti-phospho-(Thr202/Tyr204)-EKR1/2, anti-STAT3, anti-phospho-(Tyr705)-STAT3, anti-p38 MAPK, anti-phospho-(Thr180/Tyr182)-p38 MAPK, anti-mTOR, anti-phospho-(Ser2448)-mTOR, anti-p70S6K, anti-phospho-(Thr389)-p70S6K, anti-4E-BP1, anti-phospho-(Thr37/46)-4E-BP1, anti-raptor, anti-phospho-(Ser792)-raptor. Values for phosphorylated and total proteins were normalized to the loading control IR-β or Akt.

### 4.11. Cytokine Content

Tissue measurements at 15 min reperfusion (Series B) of TNFα, IL-6 and interleukin 10 (IL-10) were performed with ELISA kits (Invitrogen, Carlsbad, CA, USA) following the manufacturer’s instructions. Tissue lysates were prepared as described for Western blots and results normalized by protein content.

### 4.12. Statistical Analysis

All values are expressed as mean ± standard deviation or as median, 25–75 percentiles and range (box-and-whiskers). Statistical tests were performed for IR vs. NI, HiR MPC and LoR MPC, as well as between HiR MPC and LoR MPC. For baseline parameters, experimental groups within each perfusion series were compared, and corresponding values between perfusion Series A and B were compared. All statistical analyses were performed with GraphPad Prism 7 (GraphPad Software, Inc., La Jolla, CA, USA). Cumulative rates of glycolysis and glucose oxidation were compared using linear regressions. Kruskal–Wallis tests were employed for an overview of differences between experimental groups. Afterwards, when significant overall results were observed, comparisons between groups of particular interest at specific time points were performed with Mann–Whitney analyses. p-values were all two-tailed and adjusted for multiple comparisons (modified sequential rejective Bonferroni procedure [58]). With this approach, families of parameters, rather than individual parameters, are considered for p-value correction in order to prevent an overly severe correction [58]. To do so, three families of parameters were considered: baseline physiologic parameters, hemodynamic parameters, and biochemical parameters. Corrected p-values are reported and considered statistically significant if <0.05.

## 5. Conclusions

We investigated the heart’s endogenous metabolic and molecular conditioning mechanisms by analyzing differences between hearts that responded positively and negatively to MPC. We reported that MPC is not a safe strategy and should therefore not be applied to cardiac grafts obtained with DCD. The existence of two subgroups in our study provided the opportunity to directly compare high- vs. low- recovery hearts that were all subjected to MPC, and can thereby aid in pinpointing critical protective mechanisms, while eliminating MPC-associated changes that do not contribute to cardioprotection. Greater post-ischemic glucose uptake and glycolysis accompanied cardioprotective MPC vs. deleterious MPC, which may result from prolonged AMPK (indicated by greater raptor phosphorylation) and increased AS160 phosphorylation. Furthermore, MPC-induced cardioprotection was associated with less mitochondrial damage and a trend for lower pro-inflammatory cytokine content vs. LoR MPC (Figure 7). These aspects should help with the development and optimization of effective cardioprotective reperfusion treatments for DCD hearts. Given the pre-ischemic treatment limitations and use of *ex-situ* technology in DCD heart transplantation, controlled reperfusion strategies hold great promise to limit IRI and ensure high graft quality. Ultimately, with a widespread adoption of DCD heart transplantations, transplant activity could be increased by 15–33% [6,7] and hopefully decrease waiting list mortality.

## Figures and Tables

**Table 1:** Baseline characteristics Series A and B. BW, body weight; CF, coronary flow; CO, cardiac output; DP, developed pressure; dP/dt max, maximum first derivative of LV pressure; dP/dt min, minimum first derivate of LV pressure; HiR, high recovery; HR, heart rate; HW, heart weight; IR, ischemia reperfusion; LoR, low recovery; LV work, left-ventricular work (HR-DP product); MPC, mechanical postconditioning; NI, no ischemia. Data are expressed as mean ± standard deviation.

Variable	Series A				Series B			
	NI	IR	HiR MPC	LoR MPC	NI	IR	HiR MPC	LoR MPC
Number of hearts	7	8	11	7	6	6	7	10
BW (g)	377 ± 64	364 ± 23	350 ± 28	356 ± 26	341 ± 16	347 ± 17	373 ± 31	356 ± 23
HW (g)	1.69 ± 0.26	1.57 ± 0.09	1.64 ± 0.15	1.58 ± 0.20	1.50 ± 0.10	1.60 ± 0.20	1.75 ± 0.24	1.64 ± 0.18
LV work (mmHg ∗ beats ∗ min^−1^)	33,507 ± 1895	33,673 ± 2677	32,760 ± 2468	34,036 ± 2007	36,990 ± 1917	33,625 ± 3674	33,573 ± 5043	33,727 ± 1674
HR (beats ∗ min^−1^)	275 ± 43	266 ± 15	263 ± 16	259 ± 14	276 ± 24	264 ± 30	247 ± 38	264 ± 21
DP (mmHg)	124 ± 15	127 ± 10	125 ± 9	132 ± 6	135 ± 9	128 ± 12	136 ± 11	128 ± 8
dP/dt min (mmHg ∗ s^−1^)	−4313 ± 621	−4492 ± 991	−4173 ± 188	−4598 ± 622	−4639 ± 356	−4457 ± 906	−4797 ± 583	−5048 ± 1272
dP/dt max (mmHg ∗ s^−1^)	4354 ± 526	4475 ± 483	4361 ± 506	4621 ± 244	5046 ± 411	4246 ± 484	4673 ± 556	4498 ± 590
CO (mL ∗ min^−1^)	64 ± 14	61 ± 11	61 ± 7	63 ± 4	66 ± 6	64 ± 11	68 ± 12	65 ± 9
CF (mL ∗ min^−1^)	32 ± 5	32 ± 5	30 ± 3	32 ± 4	29 ± 4	27 ± 8	32 ± 6	33 ± 4

## Abbreviations

4E-BP1	4E-binding protein 1
ACC	acetyl-CoA carboxylase
AMPK	5′AMP-activated protein kinase
AS160	Akt substrate of 160 kDa
cTnI	cardiac troponin I
Cyt c	cytochrome c
DCD	donation after circulatory death
DP	developed pressure
dP/dt max	maximum first derivative of left ventricular pressure
dP/dt min	minimum first derivative of left ventricular pressure
Erk1	extracellular signal-regulated kinase 1
GSK3β	glycogen synthase kinase 3 beta
H-FABP	heart-type fatty acid binding protein
HiR	high recovery
HR	heart rate
IL-6	interleukin 6
IL-10	interleukin 10
IRβ	insulin receptor β
IRI	ischemia reperfusion injury
LoR	low recovery
LV work	left ventricular work
MPC	mechanical postconditioning
mPTP	mitochondrial permeability transition pore
mTOR	mammalian target of rapamycin
O_2_C	cardiac oxygen consumption
p38 MAPK	p38 mitogen-activated protein kinase
p70S6K	ribosomal protein S6 kinase
PI3K	phosphoinositide 3-kinase
raptor	regulatory associated protein of mTOR
RISK	reperfusion injury salvage kinase
SAFE	survivor activating factor enhancement
STAT3	signal transducer and activator of transcription 3
TNFα	tumor necrosis factor alpha
